# Microstructure and Mechanical/Hydrophilic Features of Agar-Based Films Incorporated with Konjac Glucomannan

**DOI:** 10.3390/polym11121952

**Published:** 2019-11-27

**Authors:** Dongling Qiao, Wenyao Tu, Lei Zhong, Zhong Wang, Binjia Zhang, Fatang Jiang

**Affiliations:** 1 Glyn O. Phillips Hydrocolloid Research Centre at HBUT, School of Food and Biological Engineering, Hubei University of Technology, Wuhan 430068, Hubei, China; qdttkl@163.com (D.Q.); twy1073981169@163.com (W.T.); WZZZZZ1996@163.com (Z.W.); 2 Department of Chemical Engineering, Guangxi Key Laboratory Cultivation Base for Polysaccharide Materials and Modifications, Guangxi University for Nationalities, Nanning 530008, Guangxi, China; leiwin@gmail.com; 3 Group for Cereals and Oils Processing, College of Food Science and Technology, Key Laboratory of Environment Correlative Dietology (Ministry of Education), Huazhong Agricultural University, Wuhan 430070, Hubei, China; 4 Faculty of Engineering, University of Nottingham, Nottingham NG7 2RD, UK

**Keywords:** agar-based film, konjac glucomannan, structural features

## Abstract

Different characterization methods spanning length scales from molecular to micron scale were applied to inspect the microstructures and mechanical/hydrophilic features of agar/konjac glucomannan (KGM) films prepared under different drying temperatures (40 and 60 °C). Note that the lower preparation temperature (40 °C) could increase the strength and elongation of agar/KGM films at high KGM levels (18:82 *wt/wt* KGM-agar, or higher). This was related to the variations in the film multi-scale structures with the increment of KGM content: the reduced crystallinity, the increased perfection of nanoscale orders at some KGM amounts, and the negligibly-changed morphology and molecular chemical structure under 40 °C preparation temperature. These structural changes initially decreased the film tensile strength, and subsequently increased the film strength and elongation with increasing KGM content. Moreover, under the higher drying temperature (60 °C), the increased KGM content could concurrently reduce the strength and elongation for the films, associated with probable phase separations on nano and smaller scales. In addition, the increased KGM amount tended to make the film more hydrophilic, whereas the changes in the film structures did not dominantly affect the changing trend of hydrophilicity.

## 1. Introduction

Increasing concerns on environment issues, resulting from petroleum-based plastics, have inspired individuals to develop biodegradable materials for packaging and other applications. Biopolymers, such as starch, protein, and agar, have been recognized as the promising resources for biodegradable packaging materials, because of the eco-friendly and renewable nature as well as the abundant availability [1,2,3]. However, the biopolymer-based materials probably show limitations such as poor mechanical properties and, thus, limited applications for food packaging. Numerous attempts, such as blending, lamination, surface coating, and nanotechnology, have been made to improve the performance of associated materials [4,5,6,7]. Among the methods, the blending of biopolymers with varied properties has been applied, due to the economic incentives and the combined advantages of blend systems compared with the individual components [4].

Agar, derived from red algae, is a hydrophilic polysaccharide and is mainly comprised of 1,3-linked β-*D*-galactopyranose and 1,4-linked 3,6-anhydro-α-*L*-galactopyranose or agarobiose [8]. As one typical renewable biopolymer, agar has been used in the design and development of biodegradable packaging films, considering its inherent, excellent film-forming capability and biocompatibility [9]. Nevertheless, the pure agar film is often brittle in nature and has relatively limited mechanical performance [10]. Hence, plasticizers such as glycerol need to be added to weaken the inter- and intromolecular forces between agar chains to reduce the brittleness of pure agar film.

In addition, research has revealed that β-1,4 glycosidic bond possesses higher rigidity than α-1,4 linkages, as the former linkages have very limited conformation transitions under external forces [11]. Thus, we hypothesize that polysaccharides linked by β-1,4 bonds can act as rigid components in the related blend material systems. Konjac glucomannan (KGM), extracted from the tubers of the amorphophallus konjac, is a nonionic linear natural polymer polysaccharide comprised of glucose and mannose units at a molar ratio of 1:1.6 by β-1,4 glycosidic bonds [12].

Following this hypothesis, konjac glucomannan (KGM), with glucose and mannose units by β-1,4 glycosidic bonds and excellent film-forming ability, was used to tailor the mechanical properties of agar-based film. Until now, the performance of agar-based films has been regulated by nanoparticles [13,14], nanoclays [15,16], nanocellulose [17,18], and other materials such as lignin [10]. However, to date, there have been limited investigations regarding the effects of the microstructures and practical features (e.g., mechanical properties and hydrophilicity) of agar-based films as tailored by the incorporation of KGM with β-1,4 linkages.

To this end, KGM component will be blended with agar to prepare agar/KGM binary composite films under different drying temperatures (40 and 60 °C). How the KGM amount and the preparation temperature tailor the microstructures and the mechanical/hydrophilic features of the agar-based films will be rationalized by using combined characterization techniques spanning multiple length scales. Then, the underlying structure property links will be discussed based on the evolutions in the multiscale structural characteristics (involving molecular chemical structure, nanoscale structure, crystalline structure, and micron-scale morphology) and in the KGM amount. The data from the present work would facilitate the development of agar-based films with associated performance for food and nonfood applications.

## 2. Materials and Methods

### 2.1. Materials

Konjac glucomannan (KGM) with a molecular weight (*M*_w_) of 9.67 × 10^5^ Da and Agar with gel strength of 750–1000 g/cm^2^ were purchased from Hubei Konson Konjac Technology Co., Ltd. (Ezhou, Hubei, China) and Biofroxx (Einhausen, Hessen, Germany), respectively. Glycerol was commercially supplied by Sinopharm Chemical Reagent Co., Ltd. (Shanghai, China).

### 2.2. Preparation of Agar/KGM Films

Agar/KGM binary films with different agar KGM ratios (100:0, 94:6, 88:12, 82:18, and 76:24 *wt/wt*) (coded as a_100_/K_0_, a_94_/K_6_, a_88_/K_12_, a_82_/K_18_, and a_76_/K_24_) were prepared using a casting method. The total content of KGM and agar was kept as 2.5% (*w/w*). Compositions of KGM/agar binary films are listed in Table S1 in Supplementary Materials. The film-forming solutions were prepared by dissolving 2.5 g of agar and KGM at different ratios in 100 mL of distilled water in a three-necked flask with continuous stirring at 600 rpm and 90 °C for 1.5 h. A total of 1 g of glycerol was used as the plasticizer. Then, the blend solutions (50 ± 0.05 g) were carefully casted onto a round plastic plate (radius: 15 cm), followed by drying in an oven at different temperatures (40 and 60 °C). To achieve a similar polymer mass for each agar/KGM film, the total amount of film-forming solution cast onto the plastic plate for one piece of film was controlled in the range of 50 ± 0.05 g. The dried films were collected and conditioned at 25 ± 1 °C and 57 ± 2% relative humidity before further analyses. Since the film-forming solution at the 2.5% KGM concentration (polymer concentration for the films) has very high viscosity and could not be used for casting, the pure KGM film was not provided. In the following, the code as a_100_/K_0_-40 will be used, where ‘40’ means the temperature used for the drying process. The thickness for the film samples was in the range of 60–95 μm.

### 2.3. Scanning Electron Microscopy (SEM)

According to an earlier method with modifications [19], a SEM system (JSM 6390LV, JEOL, Tokyo, Japan) was used to observe the fracture surface of the film samples. The instrument was operated at an accelerating voltage of 15 kV. Before observation, all the films were frozen in liquid nitrogen and then broken. The obtained fracture surfaces were coated with gold under vacuum for SEM observations.

### 2.4. Attenuated Total Reflectance Fourier-Transform Infrared Spectroscopy (ATR-FTIR)

A Nicolet iS10 (Thermo Fisher Scientific, Madison, Wisconsin, USA) spectrometer, with a Nicolet Smart Orbit ATR (Thermo Fisher Scientific, Madison, Wisconsin, USA) accessory and a diamond internal reflection element, was used to detect the ATR-FTIR spectra of the film samples. The spectra were collected at a resolution of 4 cm^−1^ in the range of 4000–400 cm^−1^ for a total of 32 scans. The air spectrum was used as the background and then subtracted from the spectrum of each film sample.

### 2.5. Small Angle X-Ray Scattering (SAXS)

Following our method with modifications [20], the BL19U2 SAXS beamline at Shanghai Synchrotron Radiation Facility (Shanghai, China) was used to collect the SAXS data for the film samples. The films were placed on a sample stage from the facility. The scattering data were collected using a Pilatus 1 M camera with a testing time of 10 s. The scattering of empty cell was the background. The background was subtracted from the sample data, and then the background-subtracted data were normalized. The data at q values of about 0.01 to 0.50 Å^−1^ were used. As defined previously [21], the scattering vector, q, was equal to 4 π sinθ/λ in which 2θ represents the scattering angle, and λ means the wavelength of incident light.

### 2.6. X-ray Diffraction

The crystalline structure of the films were inspected using a D8 Advance X-ray diffractometer (Bruker, Madison, Wisconsin, USA) operated at 40 kV and 30 mA, adapted from an earlier method with alterations [22]. The XRD curves were recorded for a 2θ range of 4–40° under 0.02° step size at a step rate of 0.5 s per step.

### 2.7. Mechanical Properties

According to the ASTM–D–882–91 standard method (ASTM–D–882–91, 1991), the mechanical measurements were performed on a Texture Analyzer (TA. XT Plus, Surrey, UK). The films were cut into strips of 5 mm × 50 mm and clamped between grips to determine the tensile strength (σ_t_) and elongation at break ($\varepsilon_{b}$). The initial grip length was 50 mm and the cross-head speed was 0.5 mm/s. The curves of force (N) versus deformation (mm) were recorded using the Texture Expert software (6.1.16.0, TA. XT Plus, Surrey, UK). The film thickness (μm) was measured by a micrometer (Shanghai Liuling Instrument Company, Shanghai, China). The values of σ (MPa) and $\varepsilon_{b}$ (%) were calculated using Equations (1) and (2) respectively.

σ_t_ = F/T × W (1)

(2) $$\varepsilon_{b}\;=\;(L\;-\;L_{0})/L_{0}\;\times \;100\%$$

where *F* (N) indicates the maximum force; *T* (cm) or *W* (cm) represents the thickness or width of the film; *L*_0_ (cm) reflects the original film length; and *L* (cm) means the length of stretching film.

### 2.8. Contact Angle Analysis

Using a method described previously [23], the air/liquid contact angles of water (H_2_O) and diiodomethane (CH_2_I_2_) on the surface of each agar/KGM composite film were determined using a contact angle goniometer (OCA15EC, Dataphysics, Filderstadt, Germany) with the sessile-drop method. Each of the films were cut into strips and placed on the horizontal movable stage. A total of 2.0 μL of deionized water or 0.7 μL of CH_2_I_2_ was dropped on the surface of the film using a micro-syringe. The measurements were taken every 1 s to determine the equilibrium state of water or CH_2_I_2_ droplet on the film surface. For the samples, the contact angle of water on agar/KGM composite films changed gradually as time rose due to the hydrophilicity nature of agar-based films; the contact angle of CH_2_I_2_ showed less significant alterations. Here, the contact angle of H_2_O or CH_2_I_2_ at the time point of 30 s was chosen to indicate the wettability of agar-based films. The total surface energy for each sample was calculated using the software SCA20 version 5.0.38 (Dataphysics, Filderstadt, Germany).

### 2.9. Statistical Analysis

Data were expressed as means ± standard deviations (SD). A statistical difference of *p* < 0.05 was considered to be significant. Statistical analysis was carried out in Microsoft Excel 2010 (Redmond, WA, USA).

## 3. Results

### 3.1. Fracture Surface Morphology

**Figure 1:**
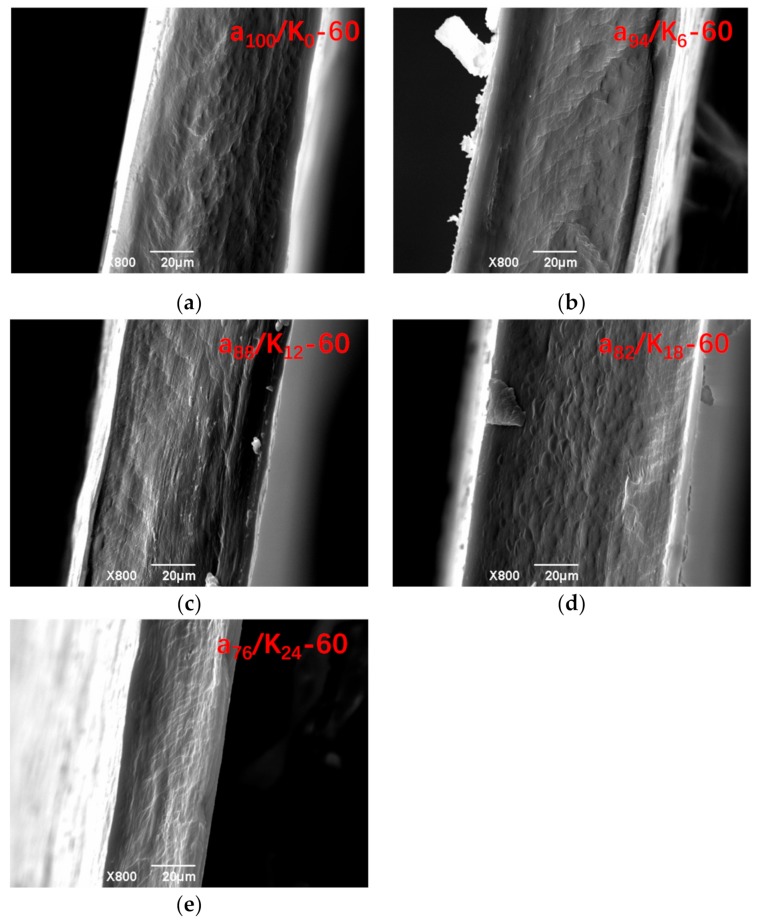
SEM photos of agar (**a**) and agar/KGM films (**b–e**) prepared at drying temperature of 60 °C.

Figure 1 includes the SEM photos for the fracture surface of agar and agar/KGM binary blend films. All of the films prepared with the drying temperature of 60 °C presented a smooth and continuous fracture surface without visible micron-scale features of phase separation. The films prepared at the lower drying temperature (40 °C) displayed a similar fracture surface (not shown). This suggests that the agar and KGM components within the films showed good miscibility.

### 3.2. FTIR Analysis

**Figure 2:**
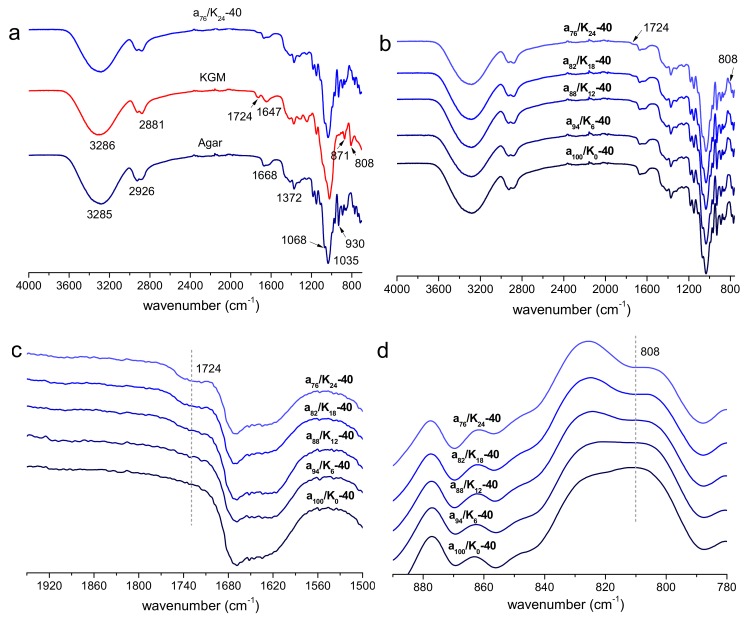
FTIR spectra of agar and KGM (**a**), and agar/KGM films (full, (**b**); enlarged, (**c**) and (**d**)) prepared under drying temperature of 40 °C.

The FTIR spectra of agar, KGM, and agar/KGM films are shown in Figure 2 and Figure S1 in Supplementary Materials. The agar film exhibited a broad absorption band at about 3285 cm^−1^ ascribed to the stretching of –OH group, and a peak at about 2926 cm^−1^ due to the C–H stretching related to the ring methine hydrogen bond in agar [24]. The peak at about 1668 cm^−1^ indicated the stretching vibration of the conjugated peptide bond formed by amine (–NH) and acetone (–CO) groups [25]. Additionally, the peak at about 1372 cm^−1^ confirmed the presence of the ester sulfate group, while the peaks at around 1068, 1035, and 930 cm^−1^ corresponded to the C–O stretching of 3,6-anhydro-galactose. For the KGM film, the bands at 3286 and 2881 cm^−1^ could be attributed to the stretching of the –OH group and the C–H of methyl in KGM, respectively. The stretching peaks at around 1724 cm^−1^ was due to the acetyl groups. The broad peak at 1647 cm^−1^ was assigned to the stretching of C–O of hydroxyl groups. The peaks at about 871 and 808 cm^−1^ were related to the mannose in KGM [26]. For the agar/KGM films (Figure 2b and Figure S1 in Supplementary Materials), all the samples displayed characteristic absorption peaks from both agar and KGM components. Expectably, a higher content of KGM made its characteristic peaks at about 1724 and 808 cm^−1^ more apparent (Figure 2c,d). Moreover, the positions of IR peaks for the agar/KGM blend films were similar to those for the control films (nonblended agar films) (see Figure 2b and Figure S1 in Supplementary Materials), revealing negligibly changed chemical structures of agar and KGM molecules under the selected drying temperatures of 40 and 60 °C.

### 3.3. Nanoscale Structural Features

**Figure 3:**
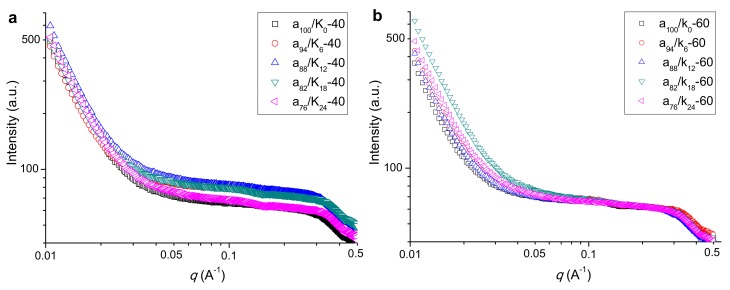
Small angle x-ray scattering (SAXS) plots for agar and agar/KGM films prepared at drying temperatures of 40 °C (**a**) and 60 °C (**b**).

The SAXS patterns for agar, KGM, and agar/KGM films prepared at different temperatures are included in Figure 3. Under 40 °C preparation conditions, the films showed a broad shoulder centered at about 0.3 Å^−1^, reflecting the presence of molecular orders on the nanoscale [19,27]. This result, with the XRD results, affirms that the crystallites could form within amorphous materials, distributed on nanoscale range. Compared to the agar film (a_100_/K_0_-40), the a_94_/K_6_-40 film possessed almost unchanged intensity at *q* < about 0.03 Å^−1^ and increased intensity at *q* > about 0.03 Å^−1^. That is, the addition of KGM in the blend film could increase the electron density (compactness) difference between the ordered and the amorphous regions, presumably due to the increased perfection of ordered regions on smaller scales (length scale below 20 nm) corresponding to *q* > about 0.03 Å^−1^. The increase of KGM content (up to 82:18 *wt/wt* agar:KGM) did not further enhance the scattering intensity at *q*-values above 0.03 Å^−1^. However, when an even higher amount of KGM was used, the a_76_/K_24_-40 film displayed decreased scattering intensity at *q* > approximately 0.03 Å^−1^ and eventually a similar whole scattering pattern to that of the neat agar film. Hence, this high KGM content made the film more amorphous (confirmed by XRD), allowing a similar electron density difference between ordered and amorphous regions to that for the neat agar film on the whole detected nanoscales.

With higher drying temperature (60 °C), there was also a broad shoulder peak for the agar and agar/KGM films, clearly revealing the existence of certainly-distributed molecular organizations on the nanoscale. Unlike the case at a lower drying temperature (40 °C), the addition of KGM with the increased amount was hard to induce changes in the scattering intensity at *q* > about 0.03 Å^−1^ (i.e., shoulder peak range); but the agar/KGM films exhibited more intense scattering at *q* < about 0.03 Å^−1^ than did the agar film (a_100_/K_0_-60). The value of *q* has a reciprocal relation to the length scale. Hence, the higher drying temperature (60 °C) contributed to the occurrence of structural changes on larger scales than did the lower drying temperature (40 °C).

### 3.4. Crystalline Structure

**Figure 4:**
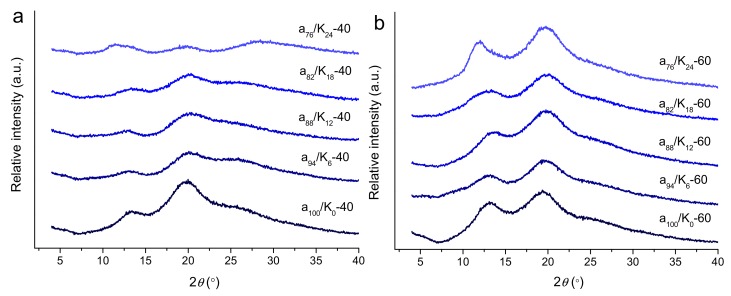
XRD curves of agar and agar/KGM films prepared at drying temperatures of 40 °C (**a**) and 60 °C (**b**).

The XRD patterns for the agar and agar/KGM films are presented in Figure 4. The agar film prepared at 40 °C showed two characteristic diffraction peaks at 13.2° and 19.8°, confirming the present of crystalline components in the agar film [25]. That is, during the film preparation, the agar was hydrated to form random coils in the film-forming solution; while the solution was dried to form a film, the discrete random agar coils could assemble to form helices that subsequently constructed the crystallites [28]. Such chain assembly manner into helices and then crystallites is also found for other biopolymers such as starch [29].

The incorporation of KGM altered the features of the film crystalline structure. The pure KGM showed sharper XRD peaks than pure agar [30]. Under 40 °C drying conditions, the agar/KGM composite films showed broad diffraction peaks with reduced intensities relative to those of the agar film. This indicates that KGM could restrict the realignment of agar molecules in the drying process, since the KGM chains probably interacted with the agar chains and eventually weakened the interactions of agar–agar chains. Such suppression effect became more prominent for the blend film at a high KGM content (a_76_/K_24_-40). Furthermore, when a higher drying temperature (60 °C) was used, the pure agar film also showed intense diffraction peaks at 13.8° and 19.8°. Unlike that for 40 °C drying conditions, the use of KGM did not substantially reduce the intensities of the two diffraction peaks, indicative of weakened suppression effect of KGM on the crystallite-forming ability of the agar/KGM films. This should be related to the accelerated removal of water solvent from the film-firming solution (thus, the crystallization of molecular chains) and the increased mobility of agar and KGM chains at a higher drying temperature (60 °C). Such accelerated crystallization and enhanced chain mobility would facilitate the ordering of agar chains (with same chemical structure) during the drying, and did not provide enough time for the interactions between agar and KGM chains. The underlying mechanism may need further investigations.

### 3.5. Mechanical Properties

**Figure 5:**
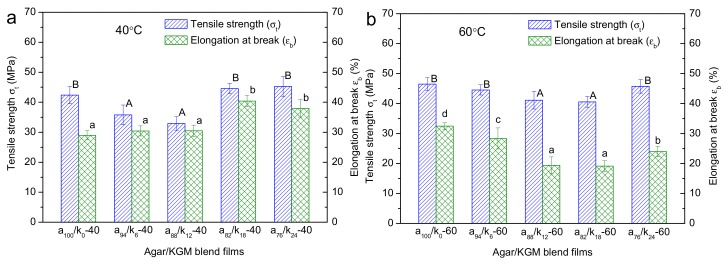
Tensile strength (σ_t_) and elongation at break ($\varepsilon_{b}$) for agar and agar/KGM films prepared with drying temperatures of 40 °C (**a**) and 60 °C (**b**). Different uppercase letter or lowercase letter on the column for tensile strength and elongation at break, respectively, differ significantly (*p* < 0.05), and lowercase letter above each column.

**Figure 6:**
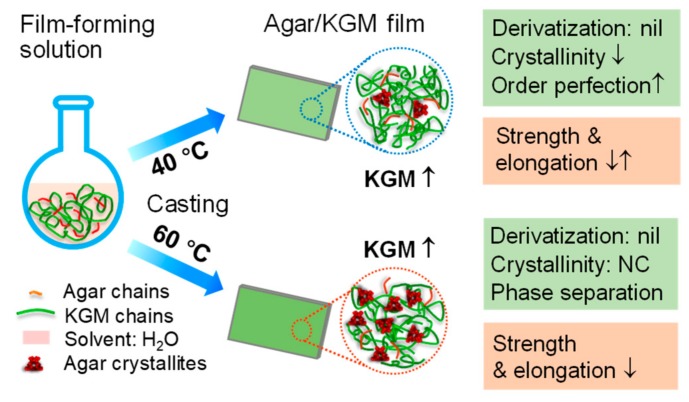
Schematic diagram for the structure−property links of agar/KGM films.

The tensile strength (σ_t_) and elongation at break ($\varepsilon_{b}$) for agar and agar/KGM binary films are displayed in Figure 5. With drying conditions at 40 °C, the initial increase of the KGM amount (up to 88:12 *wt/wt* agar:KGM) led to a reduction in the σ_t_ value and a negligible change in the $\varepsilon_{b}$; however, a further increase in the KGM proportion could induce concurrent increases in the values of σ_t_ and $\varepsilon_{b}$. Note that the existence of crystallites in a film can imped the molecule mobility within the film matrix, increasing the strength and rigidity of the film but reducing its stretchability [31]. Additionally, the pyranose polysaccharides with β-1,4 linkages are more rigid than the ones with α-1,4 linkages, due to the more difficult conformation transition under external forces [11]. Here, when a small amount of KGM emerged in the composite films (such as a_94_/K_6_-40 and a_88_/K_12_-40), the reduced content of crystalline components, with increased perfection reflected by nanostructural features, tended to somewhat decrease the strength of the films (Figure 6); and the KGM chains, without substantial derivatizations affirmed by FTIR, were too few to strengthen the films and increase their extensibility. In this case, the reduced σ_t_ and the almost constant $\varepsilon_{b}$ could be seen. Nonetheless, under higher KGM contents, while the fewer crystallites also reduced the film strength, the KGM chains distributing in the film matrices were enough to increase not only the strength but also the elongation for the composite films. These combined effects eventually increased both σ_t_ and $\varepsilon_{b}$ as shown above.

Furthermore, under the higher temperature (60 °C), the increased KGM content in general caused reductions in σ_t_ and $\varepsilon_{b}$ for the films. Nonetheless, the increased amount of crystallites (reflected by stronger XRD peaks at 60 °C than at 40 °C) and the present of nanoscale orders (shown by a similar SAXS shoulder at 40 and 60 °C) did not dominantly enhance the mechanical performance of the films prepared under 60 °C. This phenomenon should be related to the weakened KGM-agar interactions for the films prepared under 60 °C. Specifically, the films prepared at 60 °C showed negligibly weakened agar crystalline peaks with the addition of KGM, but the films acquired at 40 °C displayed gradually weakened agar diffraction peaks. This reveals that the agar chains in the films at 60 °C tended to assemble together, and eventually showed weaker interactions with KGM chains compared to the case for the films at 40 °C. Thus, phase separations between agar and KGM probably existed in the films prepared under 60 °C. Moreover, the X-ray scattering intensity changes at q-values below 0.03 Å^−1^ affirmed the structural aggregation or phase separations on scales above about 20 nm. Therefore, reduced strength and elongation were seen for the films obtained at 60 °C, as the KGM amount rose.

Hence, with the structural information spanning from molecular to micron scale, we have shown the mechanical features of the binary films as affected the KGM proportions and the preparation temperature. The results confirmed that the low preparation temperatures (e.g., 40 °C) with the relatively high KGM amount (e.g., above 18:82 *wt/wt* KGM:agar) were preferential for the generation of agar/KGM films with enhanced strength and flexibility (i.e., increased σ_t_ and $\varepsilon_{b}$).

### 3.6. Contact Angle Features

The contact angle and surface energy for agar and agar/KGM films are recorded in Table 1. All films could be considered as hydrophilic films since the water contact angles were below 90°. Among the films prepared at different temperatures, the two net agar films showed the highest water contact angle and the lowest CH_2_I_2_ contact angle. As the KGM content rose, there was a decrease in the water contact angle and an increase in the CH_2_I_2_ contact angle; these changing trends did not show apparent differences under the varied preparation temperatures. Such results indicate that increased amount of KGM, with higher hydrophilicity than agar, could make the agar/KGM films more hydrophilic; and the film structural evolutions on the different scales, as induced by the drying temperature, could not dominantly alter the changing manner on the hydrophilicity for the films. This fact could be also affirmed by the changes in the surface energy (γ_s_). In addition, relative to the films prepared under 40 °C, the counterpart films prepared under 60 °C were less hydrophilic, presumably associated with the relatively high crystallinity and nanoscale structural aggregations that certainly reduced the films’ water affinity.

## 4. Conclusions

Using combined analytical methods, this work shows the structures and mechanical/hydrophilic performance of agar/KGM binary films prepared under different temperatures (40 and 60 °C). With the lower drying temperature of 40 °C, the increased KGM amount led to a decrease in the crystallinity level, accompanied by increased perfection of nanoscale orders at some KGM contents as well as negligibly-changed morphology on micron scale and chemical structure of molecular chains. Such multiscale structural features, with increased KGM chains, initially caused a decrease in the film tensile strength and subsequently induced increases in both the film strength and elongation. Consequently, this lower temperature (40 °C) with certain high KGM amounts could endow the agar/KGM films with enhanced strength and flexibility. Furthermore, when the film drying temperature increased to a higher value (60 °C), the agar/KGM films tended to show concurrently reduced strength and elongation as the KGM fractions rose. This may be related to the phase separations on nano and smaller scales as well as an almost unchanged amount and nanoscale distribution of molecular orders such as crystallites. Additionally, for the hydrophilicity, the increased KGM amount played roles in making the composite film more hydrophilic, whereas the evolutions in the multilevel structures of the films did not apparently affect their hydrophilicity-changing trend. The present results should be of value in the rational design and production of agar-based composites with tailored mechanical and hydrophilic performance for food and nonfood usages.

## Figures and Tables

**Table 1:** Contact angle and surface energy (γ_s_) for agar/KGM binary blend films.

Samples	40 °C			60 °C		
	H_2_O	CH_2_I_2_	γ_s_ (mJ/m^2^)	H_2_O	CH_2_I_2_	γ_s_ (mJ/m^2^)
a_100_/K_0_	69 ± 2 ^d^	44 ± 1 ^A,B^	41	70 ± 2 ^d,e^	43 ± 1 ^A^	41
a_94_/K_6_	67 ± 1 ^c,d^	45 ± 2 ^A,B,C^	41	73 ± 1 ^e^	44 ± 1 ^A,B,C^	40
a_88_/K_12_	67 ± 3 ^c,d^	45 ± 1 ^A,B,C^	42	68 ± 1 ^d^	45 ± 1 ^A,B,C^	41
a_82_/K_18_	60 ± 1 ^a,b^	47 ± 1 ^C^	42	63 ± 0 ^b,c^	46 ± 1 ^A,B,C^	43
a_76_/K_24_	58 ± 2 ^a^	52 ± 1 ^D^	45	62 ± 1 ^a,b^	46 ± 0 ^B,C^	44

*^A^* Values with the different lowercase or uppercase letters letter differ significantly at *p* < 0.05. Lowercases and uppercases in the column were in the order from small values to large values.

## Supplementary Materials

The following are available online at https://www.mdpi.com/2073-4360/11/12/1952/s1, Table S1: Composition of KGM/agar binary blend films. Figure S1: FTIR spectra of agar/KGM films prepared under drying temperature of 60 °C.
