# Correction: Qiao et al. Microstructure and Mechanical/Hydrophilic Features of Agar-Based Films Incorporated with Konjac Glucomannan. *Polymers* 2019, *11*, 1952

**DOI:** 10.3390/polym13193352

**Published:** 2021-09-30

**Authors:** Dongling Qiao, Wenyao Tu, Lei Zhong, Zhong Wang, Binjia Zhang, Fatang Jiang

**Affiliations:** 1 Glyn O. Phillips Hydrocolloid Research Centre at HBUT, School of Food and Biological Engineering, Hubei University of Technology, Wuhan 430068, Hubei, China; qdttkl@163.com (D.Q.); twy1073981169@163.com (W.T.); WZZZZZ1996@163.com (Z.W.); 2 Department of Chemical Engineering, Guangxi Key Laboratory Cultivation Base for Polysaccharide Materials and Modifications, Guangxi University for Nationalities, Nanning 530008, Guangxi, China; leiwin@gmail.com; 3 Group for Cereals and Oils Processing, College of Food Science and Technology, Key Laboratory of Environment Correlative Dietology (Ministry of Education), Huazhong Agricultural University, Wuhan 430070, Hubei, China; 4 Faculty of Engineering, University of Nottingham, Nottingham NG7 2RD, UK

The authors wish to make the following correction to this paper [1]: in the original version of the published article, there is a mistake in Equation (1) on Page 3. It should be: σ_t_ = F/(T × W).

The authors apologize for any inconvenience caused to the readers by these changes. These changes have no material impact on the conclusions of our paper. The original article has been updated.
