# Supplementary material for: Microstructure and Mechanical/Hydrophilic Features of Agar-Based Films Incorporated with Konjac Glucomannan
Source: Polymers (Basel). 2019 Nov 27;11(12):1952. doi: 10.3390/polym11121952 (PMC6960638; doi:10.3390/polym11121952)
Supplement: Supplementary file 1 [file polymers-11-01952-s001.pdf]

polymers

MDPI

Supplementary Materials:

Table S1. Composition of KGM/agar binary blend films.

Samples	ratio of agar to KGM	content of agar (g)	content of KGM (g)	content of glycerol (g)
a_{100}/K_0-40	100/0	2.50	0	1
a_{94}/K_6-40	94/6	2.35	0.15	1
a_{88}/K_{12}-40	88/12	2.20	0.30	1
a_{82}/K_{18}-40	82/18	2.05	0.45	1
a_{76}/K_{24}-40	76/24	1.90	0.60	1

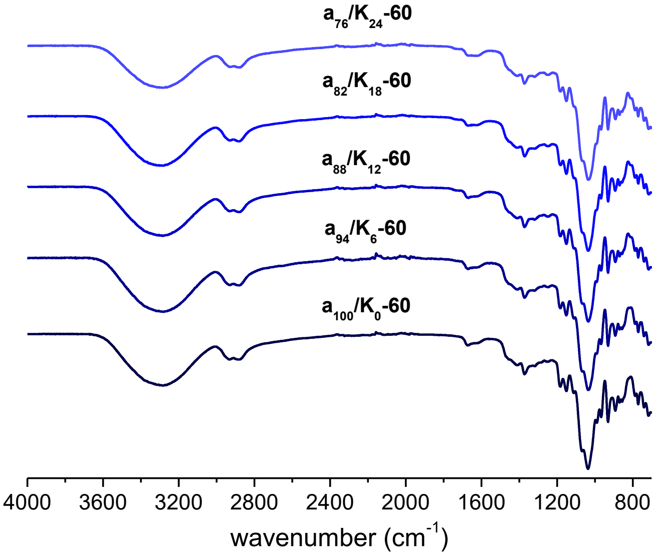

Figure S1. FTIR spectra of agar/KGM films prepared under drying temperature of 60 ºC.
